# An Evaluation of the Correlation between Hepcidin Serum Levels and Disease Activity in Inflammatory Bowel Disease

**DOI:** 10.1155/2015/810942

**Published:** 2015-01-01

**Authors:** Zehra Betül Paköz, Cem Çekiç, Mahmut Arabul, Elif Sarıtaş Yüksel, Serkan İpek, Sezgin Vatansever, Belkıs Ünsal

**Affiliations:** Department of Gastroenterology, Izmir Katip Çelebi University, İzmir Atatürk Eğitim ve Araştırma Hastanesi, Karabağlar, 35160 İzmir, Turkey

## Abstract

*Aim*. While there are many well-defined serological markers for inflammatory bowel disease (IBD), there is limited evidence that they positively affect clinical outcomes. This study aimed to evaluate the correlation between hepcidin serum levels and disease activity in IBD. *Materials and Methods*. Eighty-five consecutive IBD patients were enrolled in the study. Hepcidin serum levels were assessed using an enzyme-linked immunosorbent assay (ELISA) and were compared with disease activity as well as the interleukin-6 (IL-6) and C-reactive protein (CRP) levels. *Results*. The mean hepcidin serum levels in Crohn's disease (CD) patients in remission and in the active phase were 3837 ± 1436 and 3752 ± 1274 pg/mL, respectively (*P* = 0.613). The mean hepcidin serum levels in ulcerative colitis (UC) patients in remission and in the active phase were 4285 ± 8623 and 3727 ± 1176 pg/mL, respectively (*P* = 0.241). Correlation analysis between inflammatory markers and hepcidin serum levels indicated that there was no correlation between hepcidin levels and IL-6 (*P* = 0.582) or CRP (*P* = 0.783). *Conclusion*. As an acute-phase protein, hepcidin seems to have a lower efficacy than other parameters in the detection of activation in IBD.

## 1. Introduction

Anaemia is prevalent in inflammatory bowel disease (IBD) due to gastrointestinal bleeding and nutritional deficiencies (iron, folic acid, and vitamin B12) as a result of inadequate intake and/or absorption and chronic inflammation [1]. Hepcidin is an antimicrobial peptide that is mainly produced in the liver, and it is involved in the regulation of iron metabolism. Hepcidin binds to membrane-localised ferroportin on enterocytes, which prevents the enterocyte from releasing iron into the capillary circulation. Therefore increased hepcidin serum levels may result in hypoferritinemia [2]. Hepcidin plays a crucial role in the pathogenesis of chronic disease-related anaemia which is also called inflammation-related anaemia [3]. The hepcidin levels increase during inflammation primarily as a result of increased “bone morphogenetic protein-6” (BMP-6) and interleukin-6 (IL-6) levels [4, 5]. However, changes in the hepcidin serum levels in IBD and the correlation between hepcidin and disease activity are not fully understood. This study aimed to determine the hepcidin serum levels in ulcerative colitis (UC) and Crohn's disease (CD) as well as establish the correlation, if any, between disease activity and hepcidin levels.

## 2. Materials and Methods

This study included 85 IBD patients from the inpatient service and outpatient clinic of the Gastroenterology Department of Izmir Atatürk Training and Research Hospital between January 2012 and January 2013. All 85 patients were diagnosed with IBD based on clinical, endoscopic, and histopathological findings.

The Montreal classification was used to determine the sites of the intestinal involvement [6]. To determine whether the clinical course was remission or activation, the Mayo Clinic score (remission ≤ 2, active ≥ 3) and Crohn's disease activity index (CDAI) (remission < 150, active ≥ 150) were used for the UC and CD patients, respectively [7, 8]. Anemia was defined as a decline in blood haemoglobin to a concentration of 12 g/dL (120 g/L) in women and 13 g/dL (130 g/L) in men.

The exclusion criteria included a history of malignancy, chemotherapy, or radiotherapy; renal failure; hepatic disease; haematological or autoimmune disease; pregnancy; the presence of active infection; and current iron treatment.

The subjects fasted overnight. Blood samples for assessment of haemoglobin (Hb), C-reactive protein (CRP), hepcidin, IL-6, and ferritin were obtained at 8 a.m. in the fasting state, centrifuged at approximately 3000 rpm for 10 minutes, and stored at −80°C until use.

IL-6 was analysed with the DiaSource IL-6 EASIA (Lot number KAP1261) immunoassay method, whereas hepcidin was analysed with the enzyme-linked immunosorbent assay (ELISA) method, Uscn Life Science Inc. for Human Hepcidin (Cat. number E91979Hu). CRP was assessed with an immunoturbidimetric method using the Abbott (Lot number 81067HW00) kit in the Abbott C16000 device.

Regarding the statistical analysis, the distribution of the data and the comparisons between independent groups were assessed with the Kolmogorov-Smirnov test. The Mann-Whitney *U* test was used to determine differences between two independent groups, depending on the distribution pattern. An analysis of variance was performed to assess differences between three independent groups. Bonferroni and Tukey post hoc tests were used for subgroup analysis, and Pearson correlation coefficients were calculated for continuous variables. The Pearson chi-squared and Fisher's truncated square test were used for categorical variables. Data are presented as the means and standard deviations, and *P* < 0.05 was considered significant. All statistical analyses were performed using statistical package for the social science system software (version 17.0; SPSS Inc., Chicago, IL, USA).

The institutional review board of Ataturk Training and Research Hospital approved the study. Informed consent was obtained from each patient prior to enrolment in the study.

## 3. Results

A total of 85 patients with UC or CD were enrolled (43 males); 52 had UC and 33 had CD, respectively. Sixty percent of the UC and 48% of the CD patients were in remission. The demographic characteristics of the patients are presented in Table 1.

The median Mayo score was 2 (0–12) in all UC patients, was 6 (3–12) in patients with activation, and was 1 (0–2) in patients with remission. Median CDAI was 128 (60–480) (mean/SD, 172 ± 125) in all CD patients. Median CDAI was 210 (160–480) (mean/SD, 275 ± 123) in active CD patients. Median CDAI was 80 (60–135) (mean/SD 87.8 ± 21.3) in CD patients with remission.

The evaluation of serological and biochemical parameters revealed that the Hb, ferritin, IL-6, and hepcidin levels were not different between the UC and CD patients (*P* > 0.05), while the CRP levels were higher in the CD patients (*P* = 0.047) (Table 2).

**Figure 1:**
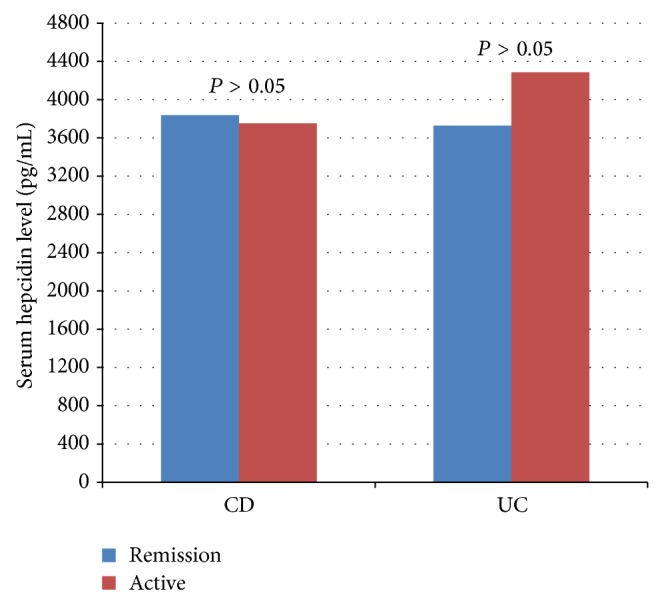
Hepcidin levels in IBD patients in remission and the active phase disease.

Among the patients with CD, there was no significant difference in the Hb, ferritin, and hepcidin levels between the patients with remission and activation (*P* > 0.05); however, the CRP and IL-6 levels were higher in those with active disease (*P* < 0.001) (Table 3). Among the patients with UC, also there was no significant difference in the hepcidin and ferritin levels between the patients in remission and activation (*P* > 0.05). However, in the patients with active disease, the Hb levels were lower (*P* < 0.001), and the IL-6 (*P* = 0.041) and CRP (*P* < 0.001) levels were higher than the levels in the patients in remission (Table 4). The serum hepcidin levels in the UC and CD patients in remission and in the active phase are shown in Figure 1.

With respect to the involvement site in patients with CD, the IL-6 (*P* = 0.261), CRP (*P* = 0.419), and hepcidin (*P* = 0.139) levels were not significantly different between the patients with ileal, colonic, and ileocolonic involvement. With respect to disease behaviour in patients with CD, the IL-6 (*P* = 0.293), CRP (*P* = 0.517), and hepcidin (*P* = 0.388) levels were not significantly different between the patients with the inflammatory, stricturing, and fistulising types. Additionally, with respect to the site of involvement in patients with UC, the IL-6 (*P* = 0.597), CRP (*P* = 0.076), and hepcidin (*P* = 0.968) levels were not significantly different between patients with proctitis, left-sided colitis, and extensive colitis.

CRP is part of the acute-phase response upon stimulation by IL-6, which makes CRP a surrogate marker for inflammation. Nevertheless, even hepcidin is also considered an acute-phase reactant; we did not find a relationship between the CRP and IL-6 levels, which significantly correlated with the clinical activation and hepcidin levels.

## 4. Discussion

Hepcidin plays a role in the regulation of iron metabolism and inflammation. Therefore, its role in inflammation-related anaemia and chronic disease-related anaemia has been thoroughly investigated. Currently, it is generally accepted that hepcidin plays a key role in inflammation-related anaemia [6]. Additionally, previous reports indicate that hepcidin is an inflammatory marker in many inflammatory processes other than inflammation-related anaemia [9, 10].

An increasing number of studies have investigated the role of hepcidin in the pathophysiology of IBD and the correlation between hepcidin and anaemia in IBD patients [11]. However, studies investigating the role of hepcidin are difficult to design because the increased levels of cytokines increase the hepatic production of hepcidin (e.g., IL-6), and many parameters affect serum hepcidin levels, such as serum iron. Few studies have assessed hepcidin in IBD, and these studies on this topic have not reported uniform results [12, 13]. The reasons for these conflicting results have not been fully elucidated. However, they have been attributed to the general inhomogeneity of the patients in the study groups with respect to disease activation or iron indexes, different numbers of patients, different age groups, and geographical and ethnic differences [14].

Although the study by Oustamanolakis et al. [13] revealed that the serum hepcidin levels and disease activity were correlated in IBD patients, we did not find such a correlation in the present study. The difference in these results may be explained by the geographical and ethnic differences, between the studies or by subjective factors, especially with regard to the determination of Crohn's disease activity index (CDAI).

Additionally, our results may be different from those of previously published studies because the latter studies did not perform subgroup analyses of serum levels of iron, and they may have used different protocols for anti-inflammatory therapy. In our study, the ferritin and haemoglobin levels in patients with active CD and in those in remission were not different, but the haemoglobin levels were significantly lower in the patients with active UC. While the serum iron levels may lead to differences in the hepatic production of hepcidin, the haemoglobin levels may also change the hepcidin production.

The study by Arnold et al. revealed that the hepcidin levels were lower in IBD patients with normal or low serum iron levels compared with a healthy population. In Arnold et al.'s study, low serum hepcidin serum levels in IBD were explained by decreased innate immunity, intestinal epithelial damage, and the loss of Paneth cells [12]. Nagy et al. reported that the serum prohepcidin levels in IBD patients were not different from those in a healthy population [15]. Semrin et al. reported that the impairment of intestinal iron absorption increased with increasing disease activity in patients with CD and that there was a correlation between disease activity and urine hepcidin levels [16].

In our study, the serum CRP and IL-6 levels were higher during active disease in both CD and UC patients. The hepcidin levels that were not correlated with the disease activity indexes were also not correlated with inflammatory markers.

We did not find any differences between the hepcidin serum levels with respect to disease localisation in UC and CD patients. Additionally, although the data were not normally distributed, the serum hepcidin levels were not different between patients with the fistulising, fibrostenotic, and inflammatory types.

Serum ferritin levels in CD patients with remission (149.5 ± 429.2) showed a remarkable discrepancy in our study, yet active CD disease patients had lower and more consistent serum ferritin levels (58.0 ± 64.7). This variation in remission might be due to multiple factors. Anemia arises in part as a result of a defect in the ability of enterocytes to transport iron and causes a net decrease in intestinal iron absorption into the systemic circulation. Small bowel is mostly effected by CD and there can be microscopic histological defects in the enterocytes while the patient is in clinical and endoscopic remission.

Consequently, the use of hepcidin alone for determining disease activity in IBD is not considered an appropriate approach. In IBD, many factors may positively or negatively affect the production of hepcidin. Based on our study, we recommend that future studies should include as many patients as possible, with equal numbers of patients in the remission and in the active stages, and that they should analyse all iron parameters that affect serum hepcidin levels, including iron serum levels, total iron binding capacity, transferrin, soluble transferrin receptor, ferritin, and haemoglobin.

## Figures and Tables

**Table 1:** Clinical and demographic characteristics of inflammatory bowel disease patients.

	Ulcerative colitis		Crohn's disease	
Age (years)	40.1 ± 12.7		47.2 ± 14.4	

	*n*	%	*n*	%

Gender				
Female	22	42	20	61
Male	30	58	13	39
Localisation of ulcerative colitis				
Proctitis	8	15		
Left-sided colitis	27	52		
Extensive colitis	17	33		
Localisation of Crohn's disease				
Ileocolonic			14	42
Colonic			6	18
Ileum			13	40
Crohn's disease				
Fistulising			5	15
Inflammatory			22	67
Stricturing			6	18
Ulcerative colitis				
Remission	31	60		
Active	21	40		
Crohn's disease				
Remission			16	48
Active			17	52

**Table 2:** Comparison of anaemia parameters, hepcidin levels, and inflammatory markers between patients with ulcerative colitis and Crohn's disease.

	Ulcerative colitis (*n* = 52)	Crohn's disease (*n* = 33)
Haemoglobin (g/dL)	12.7 ± 2.2	11.9 ± 1.9
Ferritin (ng/mL)	32.7 ± 22.0	107 ± 315.9
Interleukin-6 (pg/mL)	15.3 ± 22.8	26.8 ± 45.7
CRP (mg/dL)	1.23 ± 1.89	2.82 ± 4.45^*^
Hepcidin (pg/mL)	4090 ± 1005	3798 ± 1337

^*^ *P* = 0.047.

**Table 3:** Comparison of anaemia parameters, hepcidin levels, and inflammatory markers between Crohn's disease patients with different disease activities.

	Remission (*n* = 16)	Active disease (*n* = 17)
Haemoglobin (g/dL)	12.5 ± 1.5	11.3 ± 2.1
Ferritin (ng/mL)	149.5 ± 429.2	58.0 ± 64.7
Interleukin-6 (pg/mL)	7.4 ± 3.3	49.4 ± 60.8^*^
CRP (mg/dL)	0.44 ± 0.32	5.59 ± 5.41^*^
Hepcidin (pg/mL)	3837 ± 1436	3752 ± 1274

^*^ *P* < 0.001.

**Table 4:** Comparison of anaemia parameters, hepcidin levels, and inflammatory markers between ulcerative colitis patients with different disease activities.

	Remission (*n* = 31)	Active disease (*n* = 21)
Haemoglobin (g/dL)	13.8 ± 1.3	10.6 ± 2.1^*^
Ferritin (ng/mL)	36.7 ± 21.8	25.2 ± 21.3
Interleukin-6 (pg/mL)	10.9 ± 12.3	23.5 ± 34.1^**^
CRP (mg/dL)	0.40 ± 0.38	2.79 ± 2.55^*^
Hepcidin (pg/mL)	4285 ± 8623	3727 ± 1176

^*^ *P* < 0.001, ^**^ *P* = 0.041.
